# Significance of clinical and morphological prognostic risk factors in IgA nephropathy: follow-up study of comparison patient groups with and without renoprotection

**DOI:** 10.1186/s12882-017-0499-4

**Published:** 2017-03-14

**Authors:** Živile Riispere, Anne Kuudeberg, Elviira Seppet, Kristin Sepp, Madis Ilmoja, Merike Luman, Külli Kõlvald, Asta Auerbach, Mai Ots-Rosenberg

**Affiliations:** 1 Department of Internal Medicine, Tartu University and Tartu University Hospital, 8 Puusepa Str, Tartu, Estonia; 2 Institute of Pathological Anatomy and Forensic Medicine, Tartu University, Tartu, Estonia; 3 West-Tallinn Central Hospital, Tallinn, Estonia; 4 North-Estonia Regional Hospital, Tallinn, Estonia; 5 Department of Pathology, Tartu University Hospital, Tartu, Estonia

## Abstract

**Background:**

IgA nephropathy (IgAN) is the most frequent glomerulonephritis in many countries including Estonia. There is no specific treatment for IgAN but renoprotection is indicated when proteinuria is >1 g/day. We aimed to assess the clinicopathological correlations of IgAN and to compare the follow-up outcome of the IgAN patients receiving renoprotection with the patients with other antihypertensive regimen treatments.

**Methods:**

A retrospective kidney biopsy cohort study was carried out in consecutive 73 IgAN cases, using the new Oxford classification. The baseline and follow-up (FU, 4.1 years) clinical data were collected. The patients were divided into two main study groups according to their drug-treatment: the drug-treated and untreated patients’ groups. Two subgroups among patients receiving two different antihypertensive drugs were formed and statistically analysed: Renin-angiotensin system (RASb, renoprotection) - and calcium-channel blockers (CCB)-receiving patients. Also, patient’ subgroups with and without the presence of clinical and morphological risk factors were used for statistical analysis.

**Results:**

The patients’ mean age was 33.7 years (range 16–76). Proteinuria decreased at the end of FU (0.91 g/24 h to 0.79 g/24 h). Mean arterial pressure remained at the end of FU almost at the same level. Drug treatment was prescribed to the patients who had lower eGFR, higher proteinuria and more severe histological lesions (S1, T1/2), while the patients with minimal clinical symptoms and the ones with near-normal kidney function remained without drug treatment. The kidney function remained almost at the same normal level in untreated patients irrespective of the risk factors whereas in both treated patient’ subgroups eGFR declined. The following statistically significant correlations in the IgAN cohort were found: correlations in patients with lower kidney function (eGFR <60 ml/min/1.73 m^2^), higher blood pressure (*p* = 0.00006) and proteinuria were found irrespectively of the fact whether the patients received (*p* = 0.006) or did not receive renoprotection (*p* = 0.001). The biggest significant eGFR change by Wilcoxon rank sum test was found among the patients who had clinical and morphological risk factors and received treatment. The result was confirmed by post hoc analysis and did not depend on the presence of treatment. In the investigation of the subgroups receiving RASb we found that the lowering of eGFR did depend on the presence of clinical and morphological risk factors.

**Conclusions:**

Renoprotection is only effective in preventing the progression of IgAN when clinical and morphological risk factors are modest or missing.

## Background

IgA nephropathy (IgAN) has been found to be the most prevalent primary glomerulonephritis in many countries where the studies of kidney biopsy material have been historically performed [1, 2] or recently studied [3–6]. Therefore, numerous long-term outcome investigations with clinico-morphological assessments are available that enhance our understanding of the progression of the disease [7, 8]. The impact of morphological lesions on the outcomes in IgAN has been clarified in a number of large retrospective studies that have consistently demonstrated that the stage of the disease at presentation, as indicated by the extent of interstitial fibrosis and tubular atrophy in the biopsy, is the strongest histological predictor of renal survival [8]. Because of an asymptomatic disease course in many cases, the diagnosis of the IgAN is unfortunately obtained late, when chronic histological lesions are prominent and the renal function has deteriorated.

Clinically, IgAN symptoms and progression in individual patients are variable and the course of the disease is generally benign in cases without proteinuria, hypertension or reduced glomerular filtration rate (GFR), however, IgAN is potentially progressive to an end-stage kidney disease (ESKD). The progression of IgAN is associated with the increase of major risk factors, such as overweight/obesity, hypertension, proteinuria, smoking and severe morphological lesions (interstitial fibrosis/tubular atrophy and segmental glomerulosclerosis), which translate into a worse final outcome [7, 9].

The new Oxford classification of IgAN based on the MEST score (mesangial hypercellularity, endocapillary hypercellularity, segmental glomerulosclerosis, and tubular atrophy/interstitial fibrosis) was proposed to predict renal outcome independently from all clinical indicators at the time of biopsy and during the follow-up [10]. According to these guidelines, several studies involving large cohorts of IgAN patients have been published [11–15] and the comparisons of different IgAN validation studies have been presented [16].

We have recently presented the kidney biopsy epidemiology and the clinicopathological study based on a retrospective analysis of biopsy-proven IgAN cohort over an 11-year period in Estonia [17] with the aim to investigate clinicopathological correlations by gender, and we found that IgA nephropathy in males progresses more rapidly compared to females. Our next question arising from the previous study was to ascertain the extent of clinical and morphological risk factors having importance on the progression of IgAN and the treatment’s effect on IgAN progression. It is long known that the impairment of renal function, severe proteinuria and arterial hypertension are the strongest clinical predictors of an unfavourable outcome in IgAN [7]. And, histologically, segmental glomerulosclerosis and interstitial fibrosis/tubular atrophy are the most reliable histologic prognostic markers [18].

This is widely known that almost half of IgAN cases are asymptomatic and, for the patients with minor urinary abnormalities, the mainstay of treatment is a long-term and regular follow-up to detect renal function deterioration and hypertension [19]. Patients with IgAN especially in early stages, often do not receive specific treatment because of an asymptomatic course of the disease. Since there is no specific treatment for IgAN renoprotection (treatment with RASb) is indicated when proteinuria is >1 g/day and corticosteroids (CS) when estimated glomerular filtration rate (eGFR) is > 50 ml/min [20].

The main aim of our study was to assess the significance of both clinical and morphological prognostic risk factors in IgA nephropathy progression comparing risk factors in patient’ groups with different clinical syndromes and evaluate clinicopathological correlations. Another purpose of the study was to compare the follow-up outcome of the IgAN patients receiving renoprotection (RASb) with the patients with other antihypertensive regimen treatments.

## Methods

### Patients’ characteristics

During the period 2001–2011 years 578 native kidney biopsies were investigated at the Department of Pathology of Tartu University Hospital. IgAN constituted 35.5% of all primary glomerulopathies [6]. A total of 88 cases of IgAN during 11 years were registered. According to the recommendations of the International Consensus of IgAN study – the Oxford’s classification of IgAN [10, 21] – 73 IgAN cases were selected for the study.

The baseline clinical data were collected within 3 months from the kidney biopsy and at the end of the follow-up (FU). The medical records were reviewed with the emphasis on presenting clinical symptoms, physical examination findings, laboratory data, therapies, and outcome.

### Collection of clinical data

The following demographic data and major clinical risk factors (overweight/obesity, hypertension, proteinuria and smoking) were included for statistical analysis: data on gender and age at the time of biopsy, weight (kg), height (cm), body mass index (BMI, kg/m^2^), smoking history, presenting clinical syndrome at the time of biopsy, systolic and diastolic blood pressure (mmHg), serum creatinine (S-Creat, μmol/L), serum cholesterol (S-Chol, mmol/L), serum albumin (S-Alb, g/L). Mean arterial pressure (MAP, mmHg) was defined as diastolic pressure plus a third of the pulse pressure. Urinary protein excretion (U-prot) was expressed in grams (g) per 24 h/1.73 m^2^ in children and in g per 24 h in adults (24 h urine collection or protein/creatinine ratio). Microhematuria was ranked at the intervals of <75, 76–150, >150 erythrocytes/μL. Estimated glomerular filtration rate (eGFR, ml/min/1.73 m^2^) was calculated using the chronic kidney disease-epidemiology (CKD-EPI) formula for adults (KDIGO) and the Schwartz formula for children [22]. For the statistical analysis, the patients’ data were examined in three groups according to eGFR values as follows: 1) eGFR >90 ml/min, 2) eGFR 60–89 ml/min and 3) eGFR <60 ml/min.

### Treatment of IgAN patients

The treatment information included antihypertensive, immunosuppressive, fish oil and statins medications as well as tonsillectomy. Fish oil and the therapy with statins as well as the therapy with corticosteroids were not consistent and they have not been analysed separately in the current study. Patients with nephrotic syndrome were treated temporarily at the disease presentation with corticosteroids beside antihypertensive therapy and therefore this subgroup was not analysed separately. The data of antihypertensive drug treatment were detailed, showing the number of medications and the classes of antihypertensives: the treatment with renin-angiotensin system blockers (RASb): angiotensin converting enzyme inhibitors or angiotensin receptor blockers and the treatment with calcium channel blockers (CCB). Treatment with RASb was taken as renoprotection. 45.8% of the patients did not receive any antihypertensive and immunosuppressive drug treatment. Thus, patients were divided into two main study groups according to their drug-treatment: drug-treated and untreated patient’ groups. Two subgroups among patients receiving two different antihypertensive drugs were formed and statistically analysed: RASb-and CCB- receiving patients. Also, patient’ subgroups with and without clinical and/or morphological risk factors presence were used for statistical analysis.

### Pathomorphological data

A simplified score sheet of the Oxford classification of IgA nephropathy study was used [10]. Each biopsy was scored according to the Oxford Classification [10]: the total number of glomeruli, mesangial hypercellularity, M0/M1 (< or equivalent to >50% of glomeruli showing >4 mesangial cells in one area); endocapillary proliferation, E0/E1 (present/absent), segmental glomerulosclerosis/adhesion, S0/S1 (present/absent); glomerular membrane duplication, necrosis, cellular/fibrocellular crescent were categorized as present or absent; tubular atrophy/interstitial fibrosis, T0/T1/T2. Arteriosclerosis, A0/A1/A2, were categorized as well according severity absent/mild (0–25%), moderate (26–50%) or severe (>50%).

### Statistical analysis

The patients’ demographics, laboratory values, clinical and morphological measures were described, using the means and standard deviations (SD) for continuous variables and percentages for categorical variables. Descriptive statistics were used to characterize the cohort. The means were compared by the Student’s *t*-test, and the medians were compared using the Mann–Whitney *U* test. The continuous variables were compared using the Student’s *t*-test for independent samples, after verifying the normality of distribution, using the Kolmogorov-Smirnov test, or by the analysis of variance (ANOVA) when comparing more groups. Differences of eGFR decrease between study groups were assessed using Wilcoxon rank sum test for continuous variables.

The univariate and multivariate logistic regressions were used to examine associations between independent and dependent variables. The independent variables included: age, gender, BMI, MAP, eGFR, proteinuria, smoking history. The Spearman Rank Order Correlations were used to assess bivariate relationships between clinical variables and morphological scores. All the data were collected in a standard Excel spreadsheet and stored on a standard Excel database. The statistical analyses were conducted using the Statistics 12.0 statistical programme. The statistical significance threshold of *P* ≤ 0.05 was adopted.

## Results

### Patients’ clinical features at the time of biopsy

The patients’ demographic and clinical details are shown in Table 1. We analysed totally 73 biopsy-proven IgAN cases where 62% of patients were males and 38% females. The males were affected more often than the females (males: females = 2:1). Patients’ mean age at the kidney disease presentation, when kidney biopsy was performed, was 33.7 years (range 16–76 years). The mean time between the first clinical presentation and kidney biopsy in the cohort was 2.6 years, the mean BMI was 26.4 ± 5.2, and, 29% of all patients were smokers, male predominance occurred (23%). At the time of biopsy, a small number of patients (4%) presented with recurrent macroscopic hematuria and the main clinical syndromes were microscopic hematuria (48%) or microhematuria with proteinuria (39%). Eight patients were lost to follow-up. For the rest of 64 patients (59% male and 41% female), the mean follow-up was 48.9 months and drug treatment was prescribed for 58% of patients. The patients in the drug-treated group were significantly older (37.5 *vs* 28.8, *p* = 0.04), more likely to be overweight (27.5 *vs* 25.2, *p* = 0.02), with higher serum creatinine level (106.1 *vs* 77.2, *p* = 0.02) and had lower eGFR than the patients in untreated patient’ group (76.5 *vs* 98.8, *p* = 0.01, respectively) (Table 1). Similar clinical picture and drugs prescription was also found in the patient’ subgroups according to clinical syndrome present (Table 2). Table 3 shows that RASb were prescribed to the patients having better kidney function and lower blood pressure. The lowest kidney function was noticed in patients receiving CCBs.

**Table 1:** Demographic and clinical characteristics in the drug-treated and untreated IgAN patients at the time of kidney biopsy and at the end of follow-up

Clinical data	At the time of biopsy	At the end of follow-up		
	All cases	All cases	Drug-treated	Untreated
N	73	65	38	27
Age (years)	33.7 (16–76)	33.9 (16–76)	37.5 (16–76)	28.8 (16–46)*
Duration of follow-up (months, range)		48.9 (12–144)	46.4 (12–144)	52.8 (12–135)
BMI (kg/m^2^)	26.4 ± 5.2	27.1 ± 4.8	27.5 ± 5.4	25.2 ± 4.7*
Smokers (%)	29	27.7	31.6	22.2
Presenting clinical syndrome:				
Macroscopic hematuria (%)	4	4.6	0	11.1
Asymptomatic microhematuria (%)	48	44.6	34.2	59.3
Asymptomatic microhematuria and proteinuria (%)	39	41.5	50.0	29.6
NS (%)	7	6.2	10.5	0
Acute renal failure (%)	1	1.5	2.6	0
CKD (eGFR < 60 ml/min/1.73 m^2^) (%)	1	1.5	2.6	0
MAP (mmHg)	94.5 ± 16.7	95.2 ± 9.1	96.2 ± 9.9	93.9 ± 7.7
S-Creat (μmol/L)	93.4 ± 70.7	94.4 ± 74.4	106.1 ± 94.4	77.2 ± 15.0*
eGFR (mean, ml/min per 1.73 m^2^)	94.9 ± 30.7	85.8 ± 28.6	76.5 ± 29.1	98.8 ± 22.5**
eGFR >90 ml/min (N)	38	34	16	18
eGFR 60–89 ml/min (N)	21	18	11	7
eGFR >60 ml/min (N)	13	13	11	2
U-Prot (g/day, range)	0.91 (0–10.2)	0.79 (0–8.5)	1.0 (0–8.5)	0.5 (0–3.5)*
<1 (%)	81	79	73.7	85.2
1–3.49 (%)	11	15	13.2	14.8
>3.5 (%)	8	6	10.5	0
Microhematuria (%)	65.8	40.0	39.5	40.7

*BMI* body mass index, *CKD* chronic kidney disease, *NS* nephrotic syndrome, *eGFR* estimated glomerular filtration rate, *MAP* mean arterial pressure, *U*-*Prot* urinary protein excretion

Values are expressed as mean ± standard deviation or mean, or percent

* *p*-value < 0.05 versus Drug-treated; ** *p*-value < 0.01 versus Drug-treated

**Table 2:** Pathomorphological (MEST) and clinical data of IgAN patient subgroups with specific clinical syndromes

Presenting clinical syndrome (n, %)	At the time of biopsy							At the end of follow-up		
	M (N, %)	E (N, %)	S (N, %)	T (N, %)	eGFR (ml/min)	U-Prot (g/day)	MAP (mmHg)	eGFR (ml/min)	U-Prot (g/day)	MAP (mmHg)
Macroscopic haematuria (3, 4%, all Untreated)	2; 67	0	2; 67	0	98.9 ± 25.6	0.4 ± 0.3	84.3 ± 10.1	109.1 ± 16.3	0.4 ± 0.3	91.7 ± 10.9
Asymptomatic microhematuria (35, 48%)	20; 57	7; 20	12; 34	2; 6	105.3 ± 30.7	0.1 ± 0.1	90.6 ± 8.4	95.1 ± 22.5	0.3 ± 0.8	92.2 ± 6.2
• Drug-treated (13, 37%)	9; 69	0	5; 38	2; 15	102.8 ± 36.9	0.1 ± 0.1	92.4 ± 10.5	88.8 ± 23.4	0.5 ± 1.1	92.8 ± 6.6
• Untreated (22, 63%	11; 50	7; 32	7; 32	0	106.7 ± 27.1	0.1 ± 0.1	89.7 ± 7.2	98.8 ± 21.6	0.2 ± 0.4	91.7 ± 6.0
Asymptomatic microhematuria and proteinuria (28, 39%)	24; 86	11; 39	18; 64	3; 11	89.8 ± 20.8	0.9 ± 0.6	101.1 ± 11.2	82.1 ± 29.9	1.0 ± 1.4	97.4 ± 9.9
• Drug-treated (20, 71%)	20; 100	9; 45	14; 50	2; 7	83.0 ± 18.9	1.0 ± 0.5	102.6 ± 12.5	75.1 ± 27.9	1.0 ± 1.5	97.1 ± 10.6
• Untreated (8, 29%	4; 50	2; 25	4; 50	1; 13	106.8 ± 15.3	0.8 ± 0.6	97.3 ± 6.1	99.7 ± 28.8	1.1 ± 1.2	98.1 ± 8.5
NS (5, 7%, all drug-treated)	5; 100	4; 80	5; 100	1; 20	73.9 ± 42.5	5.9 ± 2.6	100.3 ± 10.6	67.0 ± 28.3	1.1 ± 0.7	97.5 ± 11.4

*NS* nephrotic syndrome, *eGFR* estimated glomerular filtration rate, *MAP* mean arterial pressure, *U*-*Prot* urinary protein excretion, *M* mesangial hypercellularity, *E* endocapillary hypercellularity, *S* segmental glomerulosclerosis, *T* tubular atrophy/interstitial fibrosis

**Table 3:** Clinical and pathomorphological IgAN progression risk factors in studied patients at the time of kidney biopsy and at the end of follow-up in the drug-treated patient’ group

	MAP (mmHg)	eGFR (ml/min)	U-Prot (g/day)	BMI (kg/m^2^)	S1/T1 %
At the time of kidney biopsy					
All drug-treated (*N* = 39)	100.8 ± 13.9	85.7 ± 32.8	1.3 (0–10.2)	27.6 ± 5.4	63.2
RASb (*N* = 31)	102.8 ± 14.2*	82.1 ± 32.2*	1.2 (0–10.2)*	28.4 ± 5.4	61.3
CCB (*N* = 8)	110.5 ± 16.4	49.5 ± 20.4	3.4 (0.1–10.2)	31.0 ± 6.2	87.5
At the end of follow-up.					
All drug-treated (*N* = 38)	96.2 ± 9.9	76.5 ± 28.7	1.0 (0–8.5)	27.9 ± 4.9	
RASb (*N* = 30)	95.8 ± 9.6*	73.6 ± 7.4*	1.1 (0–8.5)*	28.4 ± 4.9	
CCB (*N* = 8)	102.7 ± 15.9	44.2 ± 20.9	2.5 (0–8.5)	30.0 ± 3.3	

*RASb* renin-angiotensin system blockers, *CCB* calcium channel blockers, *MAP* mean arterial pressure, *eGFR* estimated Glomerular Filtration Rate, *M*/*F* male/female, *S*1 segmental sclerosis/adhesion, *T*1 tubular atrophy/interstitial fibrosis > 25%

Values are expressed as mean ± standard deviation or percent or range

* p-value < 0.05. The *p* value compares the RASb subgroups to the groups CCB at the time of kidney biopsy or at the end of follow-up, respectively

### Patients’ clinical characteristics after the follow-up

**Fig. 1:**
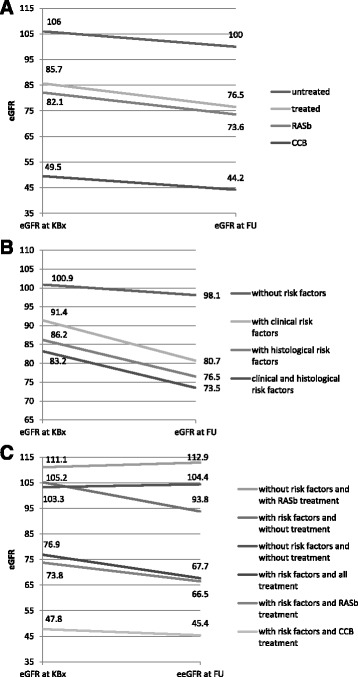
eGFR decline according to treatment regimens and IgAN progression risk factors. **a**. eGFR decline in the IgAN patient’ subgroup according to treatment regimens: untreated (*N* = 27), all drug-treated (*N* = 38) and RASb- (*N* = 31) and CCB-treated (*N* = 6). **b**. eGFR decline in the IgAN patient’ subgroups according to clinical and pathomorphological IgAN progression risk factors: without risk factors (*N* = 17), with clinical risk factors (*N* = 45), with histological risk factors (*N* = 38), with clinical and histological risk factors (*N* = 27). **c**. eGFR in the IgAN patient’ subgroups according to risk factors and the presence of treatment. eGFR, estimated Glomerular Filtration Rate; KBx, kidney biopsy, FU, follow-up

Patients in the drug-treated group were older (37.5 vs. 28.8, *p* = 0.04), more likely to be overweight (27.5 vs. 25.2, *p* = 0.02), with higher serum creatinine level (106.1 vs. 77.2, *p* = 0.02) and had lower eGFR than the untreated patients (76.5 vs. 98.8, *p* = 0.01, respectively). At the end of the FU the presenting clinical syndromes in all studied patients were similar to initial presenting syndrome but when we compared untreated and drug-treated patients important differences were found (Table 2). Macrohematuria (11.1%), microhematuria (59.3%) and asymptomatic microhematuria with proteinuria (29.6%) were the leading syndromes among untreated patients whereas among drug treated patients no macrohematuria was presented and less microhematuria was registered (34.2%) but more microhematuria with proteinuria (50.0%) were found. Among untreated patients no nephrotic syndrome or kidney failure developed whereas in many drug treated patients these clinical syndromes presented (10.5% nephrotic syndrome, 2.6% acute kidney injury, 2.6% chronic renal failure). eGFR decline was registered in all clinical and morphological risk factors having patient’ subgroups independent on the given treatment (Fig. 1c).

### Pathomorphological features

The following pathomorphology findings for each biopsy were registered: 73% had diffuse mesangial hypercellularity (M1) and 32% had endocapillary hypercellularity (E1); segmental glomerulosclerosis/adhesion was found in 52% of the cases; tubular atrophy/interstitial fibrosis (T1) was present in a few cases (10%). Arteriosclerosis (A1 and A2) was registered in 35% of cases. The lowest MEST score was present in the patient’ group having macroscopic hematuria with rather mild clinical picture (Table 2). Therefore, those patients did not receive any treatment. The IgAN patients with nephrotic syndrome had the highest MEST score (Table 2), the worst clinical picture and, therefore, all of them received the continuous treatment with antihypertensives and temporary treatment with corticosteroids. A higher MEST score was also found in patients with asymptomatic microhematuria and proteinuria whereas two thirds (71%) of those patients received the continuous treatment with antihypertensives (Table 2).

Drug treatment was prescribed to patients who had lower eGFR, higher proteinuria and more severe histological lesions (T1, S1), while the patients with minimal clinical symptoms and the ones with near-normal kidney function remained without drug treatment.

### Correlation analysis

Multiple statistically significant correlations were found in the entire IgAN study group between the MEST score components with clinical prognostic risk factors at the time of biopsy and after the FU as shown in Table 4. After analysing the patients in two study groups (drug-treated and untreated), many statistically significant correlations disappeared in the clinicopathological correlation analysis of the drug-treated patient’ group and only a few correlations remained: M correlation between U-Prot (FU); E correlation with U-Prot (FU); S correlation with eGFR (FU) and U-Prot as well as with U-Prot (FU); T correlation with S-Creat and eGFR as well as with eGFR (FU). Thus, M1, E1, S1, T1 and A2 scores were correlated with the levels of MAP, eGFR and proteinuria, being similar to the results published in the Oxford classification of IgAN study [8, 17]. A higher level of arteriosclerosis score and MEST score was noticed in those patients whose proteinuria was higher and eGFR was lower at the end of the follow-up. In the untreated patient’ group, only one statistically significant correlation between the pathomorphological and clinical risk factors was found: between arteriosclerosis and microhematuria (*r*_s_ = -0.4, *p* = 0.04).

**Table 4:** Correlation between patomorphological (MEST) and clinical prognostic risk factors

Pair of variables	Spearman R	*Enti*re group (*N* = 73) *p* value	Spearman R	*Drug*-*treated* (*N* = *39*) *p* value
M & S-Creat	0.3	0.02	-	NS
M & eGFR	−0.3	0.03	-	NS
M & eGFR (FU)	−0.3	0.01	-	NS
M & U-Prot	0.4	0.0001	-	NS
M & U-Prot (FU)	0.3	0.04	0.3	0.04
E & MAP	0.3	NS	0.6	0.03
E & S-Creat	0.3	0.02	-	NS
E & U-Prot	0.3	0.003	-	NS
E & S-Chol	0.3	0.008	-	NS
S & eGFR	−0.3	0.02	-	NS
S & eGFR (FU)	−0.4	0.0006	−0.4	0.02
S & U-Prot	0.5	0.00004	0.5	0.0006
S & U-Prot (FU)	0.4	0.0006	0.5	0.002
T & Age	0.3	0.03	-	NS
T & S-Creat	0.4	0.002	0.6	0.0002
T & MAP (FU)	0.3	0.03	-	NS
T & eGFR	−0.4	0.0009	−0.5	0.0009
T & eGFR (FU)	−0.5	0.00004	−0.5	0.001

*M* mesangial hypercellularity, *E* endocapillary hypercellularity, *S* segmental glomerulosclerosis, *T* tubular atrophy/interstitial fibrosis, *eGFR* estimated glomerular filtration rate, *MAP* mean arterial pressure, *U*-*Prot* urinary protein excretion

### Kidney function and risk factors of IgAN progression

Kidney function remained almost at the same normal level in untreated patient’ group irrespective of risk factors whereas in both treated patients’ subgroups eGFR declined (Table 3, Fig. 1a). The lowest mean eGFR at the end of the follow-up was in the patient’ subgroup who received CCBs and the highest proteinuria level was also noticed among those patients (2.5 g/24 h, range 0-8.5) comparing with other RASb treatment receiving patient’ subgroup (Table 3). The decline of mean eGFR was noticed in all study groups (Fig. 1a). The changes of eGFR in IgAN patients were found as follows: eGFR decline was noticed in 54.8% of patients, in 12.3% of the patients eGFR remained at the same level and in 30.2% of the patients it slightly rose. By ANOVA, the decline of eGFR was noticed generally in all patients’ subgroups notwithstanding which treatment they received or what kind of risk factors, clinical or morphological, they had. The Wilcoxon rank sum test confirmed that the lowest eGFR and the deepest decline were noticed in patient’ subgroups with both clinical and morphological risk factors and those patients received drug treatment (Fig. 1b). Using Mann–Whitney U comparison tests we analysed the IgAN treatment subgroups. The following statistically significant correlations in IgAN cohort were found: in patients with lower kidney function (eGFR <60 ml/min), higher MAP (*p* = 0.00006) and proteinuria was found irrespectively of the fact whether patients received (*p* = 0.006) or did not receive RASb (*p* = 0.001). Drug treatment was prescribed to patients who had lower eGFR, higher proteinuria and more severe histological lesions (T1/S1). Thus, patients with severe clinical and morphological symptoms were treated but patients with minimal clinical symptoms and with near-normal kidney function stayed without drug treatment.

According to the results of the Kolmogorov-Smirnov test, all the patients who received RASb therapy had significantly higher eGFR comparing with the other treatment receiving patients but their eGFR remained lower comparing with the untreated patient’ group (*p* < 0.005). There was no difference in the follow-up proteinuria level. Examining patients with corticosteroids treatment we did not find statistically significant differences.

The dispersion analysis showed that BP affected significantly the dispersal of eGFR (*p* = 0.000005), especially when we estimated BP concurrence with smoking (*p* = 0.01). After the FU eGFR was lower in both study groups and more significantly among the patients with clinical and morphological risk factors (ANOVA). The biggest significant eGFR change by the Wilcoxon rank sum test was found among the patients who had risk factors and received treatment. The result was confirmed by a post hoc analysis and did not depend on the presence of treatment. In the investigation of subgroup receiving RASb we found that the lowering of eGFR did depend of the presence of clinical and morphological risk factors. Finally, we assessed the study subgroups separately according only to major clinical risk factors (overweight/obesity, hypertension, smoking and proteinuria), and the following results were found: in patients with major clinical risk factors and without drug treatment the decline of eGFR was 13% from initial but in those who had major clinical risk factors and received RASb it was 10.5%. The decline of eGFR at the end of FU was the lowest (9.4%) in patients without clinical risk factors and who received RASb treatment (Fig. 1c). However, we did not find statistically significant reliability for these differences. Thus, RASb are only effective in preventing of the progression when clinical and morphological risk factors are modest or missing.

## Discussion

The current investigation is another analysis of our clinico-morphological correlation’s study [17] where we used recently published a new Oxford classification of IgAN [8]. This classification is aimed to define reproducible and useful kidney biopsy-based prognostic indicators [8]. We aimed to study the significance of both morphological and clinical risk factors in the progression of IgAN and to assess IgAN clinicopathological correlations as well. Also we aimed to compare the follow-up outcome of the IgAN patients receiving renoprotection (RASb treatment) with the patients receiving other antihypertensive regimen treatments. Our IgAN patients’ cohort consisted of patients having normal mean eGFR at the time of biopsy unlike of other similar studies where clinico-morphological correlations have recently been studied using the Oxford classification [23]. The mean eGFR of patients by the Oxford derivation [24], North American validation [25], and VALIGA studies [8] was 68.4 ml/min/1.73 m^2^ but in our cohort it was 94.9 ml/min/1.73 m^2^ meaning that we have been biopsied patients earlier comparing with other IgAN studies. Probably, the most interesting characteristics of our cohort were that almost half of the patients had only microscopic haematuria presenting very early or mild disease. In some European centres including ours and also in Japan, patients with persistent microscopic haematuria are biopsied and this gives diagnosis and a chronicity level as well to clinicians for further management of patients with early and mild disease. And, this is also a meaning of the Oxford classification – to find out a MEST score for better management of patients during a long-lasting disease course. However, this difference in patients’ cohorts comparing with other IgAN studies may give other advantages like the opportunity to assess more closely the impact of clinical and morphological risk factors on the decline of renal function in patients with early or mild disease and different treatments.

In our study, drug treatment was prescribed to the patients who had lower eGFR, higher proteinuria and more severe histological lesions (S1, T1/2). The decline of eGFR was noticed in all treated or untreated study groups. However, eGFR stayed stable in patient’ subgroups without risk factors and without treatment as well as without risk factors and with RASb treatment. As mentioned above, the mean eGFR was normal in the whole IgAN cohort and, although, it declined in the end of the follow-up, it still stayed at a near-normal level (85.8 ml/min/1.73 m^2^). IgAN patients with less clinical symptoms (lower MAP, without proteinuria, with normal kidney function) stayed without treatment but patients with severe clinical symptoms (higher MAP, microhematuria with proteinuria, lower kidney function) and serious morphological lesions (S1, T1/2) were treated. A similar management of IgAN where the drug-treatment is mostly indicated for more serious cases has also been described in many other studies [23]. The optimal approach to the treatment of IgAN is still uncertain but renoprotection is a well-known approach in the management of various chronic kidney diseases today following basic experimental [26, 27] and clinical landmark studies [28–30]. Our study revealed that RASb were generally effective in arresting the IgAN progression and diminishing proteinuria but RASb, as renoprotection, were not effective in patients with clinical and morphological risk factors present. When we assessed the study subgroups separately only according to clinical and morphological risk factors, the decline of eGFR was the lowest in RASb treated patients without clinical risk factors. Thus, maximal renoprotection can be achieved when patients’ blood pressure is normal, they do not smoke and keep their body weight within normal range. We were able to demonstrate that RASb were only effective in preventing the progression when clinical risk factors were modest or missing. The same question was raised by other authors but they failed to find similar differences in the subgroups based on treatment exposures [23].

Another aspect deserves to be pointed out. Similarly to RASb usage also CS was prescribed to IgAN patients who had proteinuria, more serious morphological lesions (S1, T1/2) and lower kidney function. In our cohort the lowest kidney function was noticed in the patients receiving CCBs or CSs. Interestingly, since KDIGO guideline now recommends CSs to patients with eGFR >50 ml/min/1.73 m^2^, we have historically used these drugs in many patients below a recommended level. Coppo et al. have also reported similar findings in VALIGA study where immunosuppression was more frequently used in the patients with lower eGFR [31]. Those findings demonstrate a nephrologists’ aggressive therapeutic attitude in treating IgAN patients with more severe CKD and, on the other hand, leaving the patients without severe clinical symptoms untreated.

This study also provides a novel insight into importance of arteriosclerosis as well as microhematuria. Namely, we observed arteriosclerosis being correlated only with microhematuria in untreated patients. Whereas this morphological lesion had no significant correlation in the drug-treated study group and microhematuria has generally been considered as a benign clinical symptom, we speculate that in the long term microhematuria may have much bigger importance than expected because the decline of renal function was still found in the untreated study group patients. Recent findings suggest a pathogenic role for glomerular haematuria in kidney disease [32]. Although the majority of studies rule out a repercussion of hematuria on the renal outcome of patients with the IgAN, the true role of hematuria on renal outcome remains uncertain [33].

We are aware of the limitations of our retrospective study which should be considered when interpreting results. At first, our cohort was small and the treatment of patients was not standardized. We did not have data on the dose of RASb and in CS-received patient’ group, also CCBs were also prescribed. Although we found the decline of eGFR at the end of the follow-up, our follow-up time may still be short considering the interpretation and comparison of data in different study subgroups.

## Conclusions

RASb are only effective in preventing the progression of IgAN when clinical and morphological risk factors are modest or missing. Clinical risk factors have important influence on long-term outcome and should be managed carefully in early stages of the disease progression. The correlation of arteriosclerosis with microhematuria may explain the fact that long-term microhematuria has much bigger importance in IgAN patients than expected because the decline of renal function was also found in the untreated patient’ group and may be associated with arterial damage through persistant microhematuria.

## Abbreviations

ANOVA: Analysis of variance
BMI: Body mass index
CCB: Calcium channel blockers
CKD-EPI: Chronic kidney disease-epidemiology
CS: Corticosteroids
eGFR: Estimated glomerular filtration rate
ESKD: End-stage kidney disease
FU: Follow-up
GFR: Glomerular filtration rate
IgAN: IgA nephropathy
MAP: Mean arterial pressure
MEST: Mesangial hypercellularity, endocapillary hypercellularity, segmental glomerulosclerosis, and tubular atrophy/interstitial fibrosis
RASb: Renin-angiotensin system blockers
S-Alb: Serum albumin
S-Chol: Serum cholesterol
S-Creat: Serum creatinine
SD: Standard deviations
U-prot: Urinary protein excretion
